# Identification of Immune Subtypes of Lung Squamous Cell Carcinoma by Integrative Genome-Scale Analysis

**DOI:** 10.3389/fonc.2021.778549

**Published:** 2022-02-02

**Authors:** Liyuan Yin, Wen Zhang, Dan Pu, Xiaoqian Zhai, Yiyun Lin, Qiang Wu, Tangel Chang, Jia Hu, Yan Li, Qinghua Zhou

**Affiliations:** ^1^ Lung Cancer Center, West China Hospital, Sichuan University, Chengdu, China; ^2^ Department of Immunology, National Cancer Center/National Clinical Research Center for Cancer/Cancer Hospital, Chinese Academy of Medical Sciences and Peking Union Medical College, Beijing, China; ^3^ Graduate School of Biomedical Sciences, University of Texas (UT) MD Anderson Cancer Center, Houston, TX, United States; ^4^ Department of Radiation Oncology, University of Toledo, Toledo, OH, United States

**Keywords:** lung squamous cell carcinoma, immune, prognosis, immune infiltration, neutrophil

## Abstract

**Background:**

Characterization of the tumor microenvironment is helpful to understand the tumor immune environment of lung cancer and help predict the prognosis.

**Methods:**

First, immune subtypes were identified by consensus subtype among lung squamous carcinoma (LUSC) patients. Immune cell infiltration was evaluated by CIBERSORT and ESTIMATE analyses. Then, based on differentially expressed genes (DEGs) identified, a risk score model was constructed. Finally, gene *FPR1* was validated by using YTMLC-90.

**Findings:**

LUSC samples were divided into four heterogeneous immune subtypes, with significantly different prognoses with subtype 4 having the poorest overall survival (OS). The immune infiltration score showed that subtype 4 was characterized as immune enriched and fibrotic, while subtype 3 was tumor enriched. DEG analysis showed that upregulated genes in subtype 4 were enriched of neutrophil and exhausted T cell-related biological processes. Based on a univariate Cox regression model, prognostic 7 immune-related genes were combined to construct a risk score model and able to predict OS rates in the validation datasets. Wound healing and transwell assay were conducted to evaluate the invasion property after activating the gene *FPR1.*

**Interpretation:**

The analysis of tumor immune microenvironments among LUSC subtypes may provide new insights into the strategy of immunotherapy.

## Introduction

Non-small cell lung cancer (NSCLC) is the leading cause of cancer-related death worldwide (1). Histologically, non-small cell lung cancer is divided into lung adenocarcinoma, lung squamous cell carcinoma (LUSC), and large cell carcinoma (2). Despite great improvement in lung cancer treatment, many patients still experience resistance to drug with poor survival rates, especially for locally advanced NSCLC (3). Thus, more effective treatments are in urgent need. Cancer immunotherapy has revolutionized the clinical management of lung cancer (4). There is growing evidence that showed the importance of immune regulation in lung cancer (5, 6). For example, immune checkpoint inhibitors (ICIs) have been approved as the first-line treatment for advanced-stage lung squamous cell carcinoma with PD-L1 expression >1% (7–9). However, the heterogeneity of the tumor microenvironment brings confusing treatment response among individual patients (10). It is documented that some patients with high PD-L1 expression do not respond to ICIs while some patients with low PD-L1 expression obtain benefits from ICI treatment. The development of more effective treatments is hindered by incomplete knowledge of the genetic determinant of immune responsiveness.

Tumors grow under an intricate regulation environment of epithelial cells, vascular and lymphatic vessels, cytokines and chemokines, and infiltrating immune cells (5). Different immune cells, such as the various types, functional polarization, and local distribution through the tumor, have been shown to influence the clinical outcome for cancer patients (5). The tumor-immune microenvironment consists of multiple immune and stromal cells as well as some immunomodulators (11). Increasing evidence has shown that the different responses of ICI treatment are related to the heterogeneity of the tumor microenvironment. Thus, characterizing the tumor microenvironment of LUSC would be helpful to understand the tumor immune environment in LUSC patients and predict prognosis of patients.

In this study, we aimed to identify the immune subtypes in LUSC and explore the potential prognostic genes among different subtypes. LUSC expression data from the Cancer Genome Atlas (TCGA) were divided into four consensus subtypes according to the immune related genes’ expression. We demonstrated that subtype 4 was characterized as immune-enriched but fibrotic with the poorest overall survival (OS). There are 7 genes upregulated in subtype 4 which correlates with neutrophil and exhausted T cell and were used to construct a risk model to validate in different datasets. Overall, these data demonstrated that the heterogeneity in LUSC and identified prognostic genes among different immune subtypes potentially plays a role in antitumor immunity.

## Materials and Methods

### Patients and Clinical Characteristics

The gene expression profile and clinical information of lung squamous carcinoma patients were obtained from the TCGA database. In addition, validation datasets GSE29013 (12), GSE67061 (13), and GSE73403 were obtained from Gene Expression Omnibus (GEO) (http://www.ncbi.nlm.nih.gov/geo).

### Data Preprocessing

For the TCGA dataset, the RSEM-normalized RNA-seq data were downloaded from the TCGA data portal. The gene expression value was log_2_-transformed for subsequent analysis. GEO raw datasets were obtained and converted to normalized data using the median scale method by the R package “limma” (14) and then used for the analysis.

### Identification of Immune Subtypes of LUSC

Immune-related genes were extracted from the Gene Ontology (GO) database by searching immune/inflammation/defense-related GO terms, obtaining 3,555 genes. The genes with no expression in RNA-Seq data from LUSC-TCGA are more than 80% of samples and were excluded in the study. 2,678 genes were used to conduct Consensus Subtype Plus analysis (15). Gene expression data were median centered. The subtyping program was performed with 1,000 iterations, by sampling 80% of samples at each iteration. The optimal subtype number was determined by cumulative distribution function curves of the consensus score. Pairwise comparisons among identified subtypes were performed by SigClust analysis (16).

### Immune Cell Abundance Identified

CIBERSORT (17) is based on a linear support vector regression that estimates the degree of immune cell infiltration. By using the CIBERSORT method, the proportion of 22 kinds of immune cells infiltrating different subtypes was identified. Twelve exhausted T cell markers and 19 immune checkpoint markers were also evaluated between the subtypes. ESTIMATE was used to calculate immune and stromal scores (18).

### Differential Analysis of Expressed Genes

For the differentially expressed genes between different subtypes, Student’s t-test was utilized to compare the samples in one subtype to other subtypes. |log FC| ≥ 2, *p* <.05 was considered as significantly differentially expressed. The GO (gene ontology) and Kyoto Encyclopedia of Genes and Genomes (KEGG) pathways were analyzed by using the DAVID tool for exploring DEG function (19). Gene Set Enrichment Analysis (GSEA) was performed as previously described (20). Correlation R package and Pearson’s test were used to evaluate the correlation between genes’ expression and immune infiltration scores.

### Survival Analysis Risk Score

To build a predictive model related to prognosis, 20 upregulated cytokine genes in subtype 4 were correlated with neutrophils to undergo a LASSO analysis. Seven genes were identified and constructed as a risk model for survival prediction. The risk model combines the expression data with their coefficient and was validated in independent GEO validation datasets.

### Cell Culture

YTMLC-90 cell lines were human LUSC cell lines purchased from the Cell Library Committee on Type Culture Collection of the Chinese Academy of Sciences, Beijing. Cells were cultured in RPMI-1640 supplemented with 10% FBS in a humidified, 5% CO_2_ atmosphere at 37°C.

### Transwell Assay

YTMLC-90 cells were treated with the indicated concentration of fMLP for 24 h. Thereafter, cells were starved in serum-free RPMI 1640 medium for 12 h at 37°C in 5% CO_2_. Cell suspensions (4 × 10^4^ in 200 μl) were then added to the upper chambers with a pore size of 8 μm (Corning) and 400 μl of complete medium to the lower chambers. After being incubated for 24 h at 37°C in 5% CO_2_, the cells in the lower chamber were stained with crystal violet and then imaged and counted under the microscope.

### Wound Healing Assay

YTMLC-90 cells were cultured in 6-well plates overnight. The next day, a straight scratch was made in the center of each well using a micropipette tip. Cells were then washed with PBS and treated with the indicated concentration of fMLP for 24 and 48 h. Cell motility was assessed by measuring the movement of the cells into the scratch area after 24 and 48 h of treatment with fMLP.

### Statistical Analysis

R software (version 3.6.2) was used to perform all statistical analyses using the Student’s t-test. To conduct a survival analysis, the Kaplan–Meier approach was used, and the subsequent findings were compared using the log-rank test. GraphPad Prism was used to perform experimental analysis. (*p-value <0.05; **p-value <0.005; ***p-value <0.0005; ****p-value <0.00005).

## Results

### Identification of Immune Subtype of LUSC

**Figure 1:**
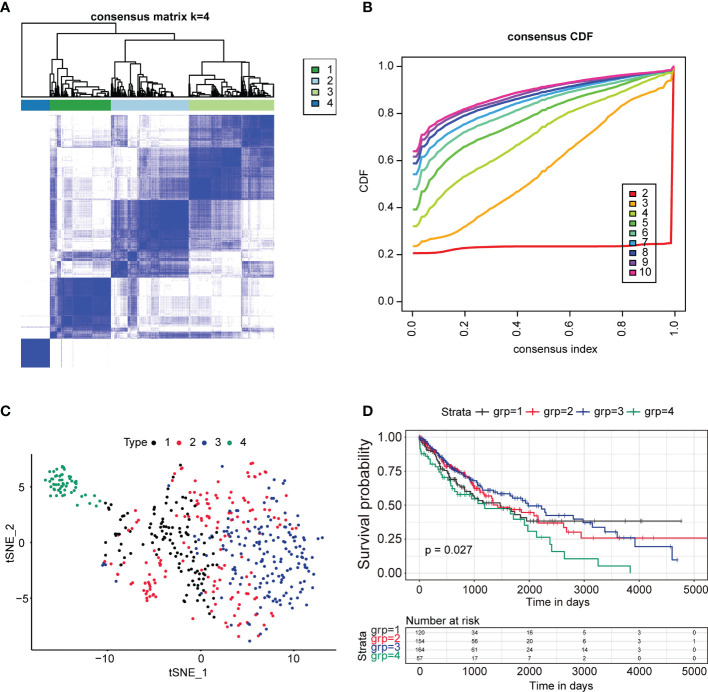
Identification of immune subtypes in lung squamous cancer patients in TCGA. **(A)** The consensus score matrix of all samples when k = 4. A higher consensus score between two samples demonstrated that they were more likely to be grouped into the same cluster in different iterations. **(B)** The cumulative distribution function (CDF) curves by consensus cluster analysis. CDF curves of consensus scores by different subtype numbers (k = 2–10) are presented. **(C)** Two-dimensional scaling plot of the expression profiles of immune-related genes in TCGA LUSC patients. Each point represents a single sample with different colors representing the four subtypes. **(D)** Survival analysis for the four subtypes of TCGA LUSC patients. The *p* value was calculated by the log-rank test. ****P < 0.0001.

To identify the immune subtype of LUSC patients, 2,678 genes related to the immune biological process were analyzed. For all TCGA LUSC patients, Consensus Subtype Plus was used to sample into k (k = 2–10) different subtypes. According to the cumulative distribution function curves of the consensus score, the optimal division was achieved when k = 4 (**Figures 1A, B**). SigClust analysis was used to analyze the difference among the four subtypes (**Supplementary Table 1**). The data showed that the consensus subtypes were significantly different among all pairwise comparisons. The distributions of the clinical parameters among the four subtypes are displayed in **Supplementary Table 2**. The two-dimensional scaling plot showed that the four subtypes were separated, with subtype four distinct from other three subtypes (**Figure 1C**). Next, we tested the prognosis of four subtypes by using TCGA-LUSC RNA-Seq data. The OS was significantly different among the four subtypes (p = 0.027, **Figure 1D**) with subtype 3 having better survival and subtype 4 being the worst.

### Landscape of the Immune Cells’ Infiltration for Four Immune Subtypes

**Figure 2:**
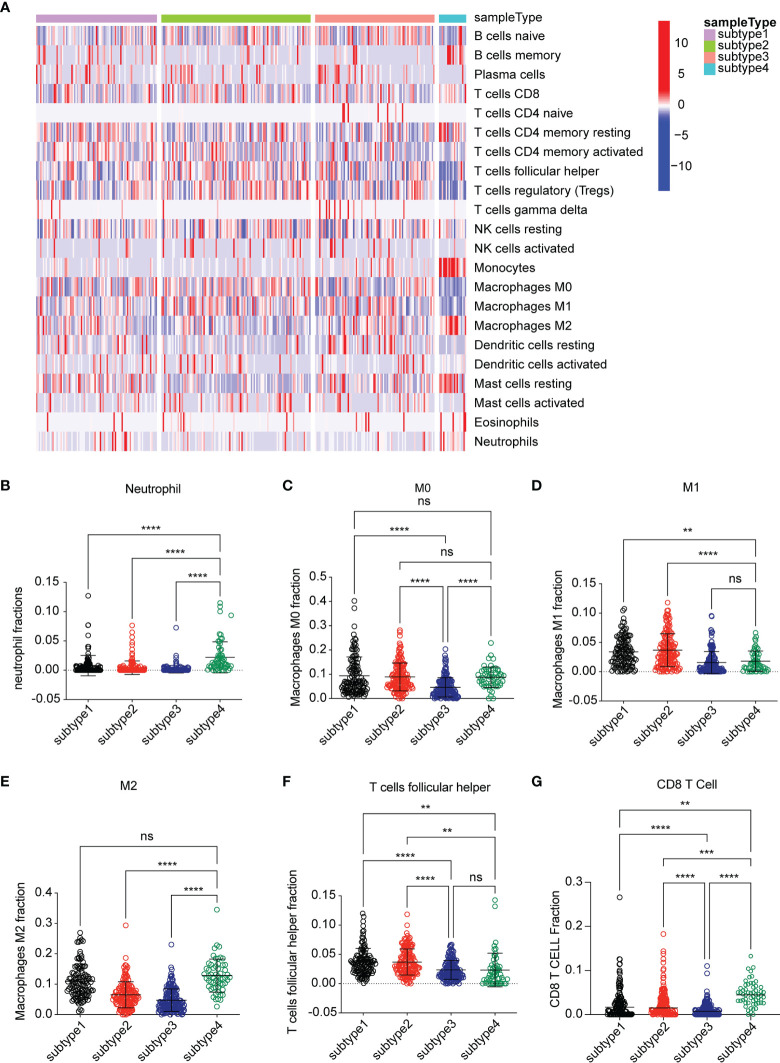
Analysis of the immune environment among the four subtypes. **(A)** Heatmap of gene expression scores of 22 immune signatures among four subtypes. Blue represented low expression. Red represented high expression. **(B–G)** The expression scores of signatures of six immune cells for four subtypes. Comparisons between two subtypes were performed by Student’s t test. *p* < 0.05 was considered significant. **P < 0.01, ***P < 0.001, ****P < 0.0001, ns, no significant.

To investigate the causes for OS difference among the four subtypes, we evaluated the difference in respect to immune cell infiltration among four subtypes by using the CIBERSORT method. The result was characterized by an abundance of infiltration of macrophage M2, neutrophil, myeloid dendritic cell activated, CD8+T cell, and CD4+ resting memory T cell in subtype 4, while subtype 3 was characterized as low abundance of infiltration of immune cells. Subtype 1 and subtype 2 were characterized as mixed infiltration of immune cells (**Figure 2A**). Based on the specific immune cell expression score, the expression signatures of M2 cell, neutrophil, and M0 cell were significantly higher in subtype 4 compared to the other subtypes (**Figures 2B, C, E**). Interestingly, the expression signature of CD8+ T cells was also higher in subtype 4 (**Figure 2G**). Follicular helper T cells were highly infiltrated in subtypes 1 and 2 compared to subtype 4 (**Figure 2F**), while CD8+ T cell, M0, M1, M2, and neutrophil cell were characterized as low infiltration in subtype 3.

### Microenvironment Analysis Among Four Subtypes

**Figure 3:**
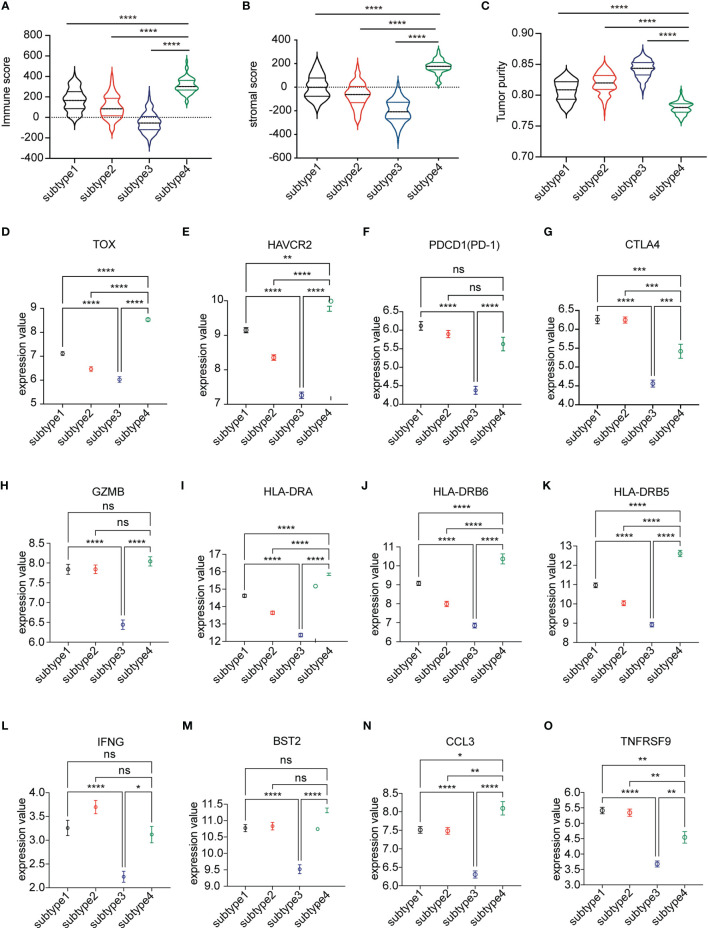
Analysis of the microenvironment among the four subtypes. **(A–C)** The immune score, stromal score, and tumor purity score evaluated among the four subtypes by ESTIMATE. **(D–O)** Differential expression of markers representing exhausted T cells in four subtypes of the TCGA LUSC cohort. The plot was presented as mean ± SEM. Comparisons between two subtypes were performed by Student’s t test. *p* < 0.05 was considered significant. *P < 0.05, **P < 0.01, ***P < 0.001, ****P < 0.0001, ns, no significant.

We next assessed the abundance of immune and stromal cell infiltration in the four subtypes. The significant differences were in subtype 3 and subtype 4 (**Figures 3A–C**). The data showed that immune score and stromal score were higher in subtype 4 than in the other subtypes, especially subtype 3 (p < 0.0001). In contrast, subtype 3 showed lower immune cell infiltration and stromal cell infiltration. We defined subtype 4 as “Immune-enriched, fibrotic” and subtype 3 as “tumor enriched”. We then analyzed the expression of T cell exhausted gene signatures which play an important role in T cell regulation and immune checkpoint genes. Interestingly, expressions of a panel of T cell exhausted gene signatures were also upregulated in subtype 4 but downregulated in subtype 3 (**Figures 3D–O**). Notably, some of the checkpoint genes showed a differential expression between subtype 3 and subtype 4. Most of the checkpoint genes were significantly upregulated in subtype 4 and downregulated in subtype 3 (**Figures 3F, G** and **Supplementary Figure 1**), which indicated that the immune environment of subtype 4 was immune suppressed and subtype 3 was not.

### Differential Gene Analysis Between Subtype 3 and Subtype 4

**Figure 4:**
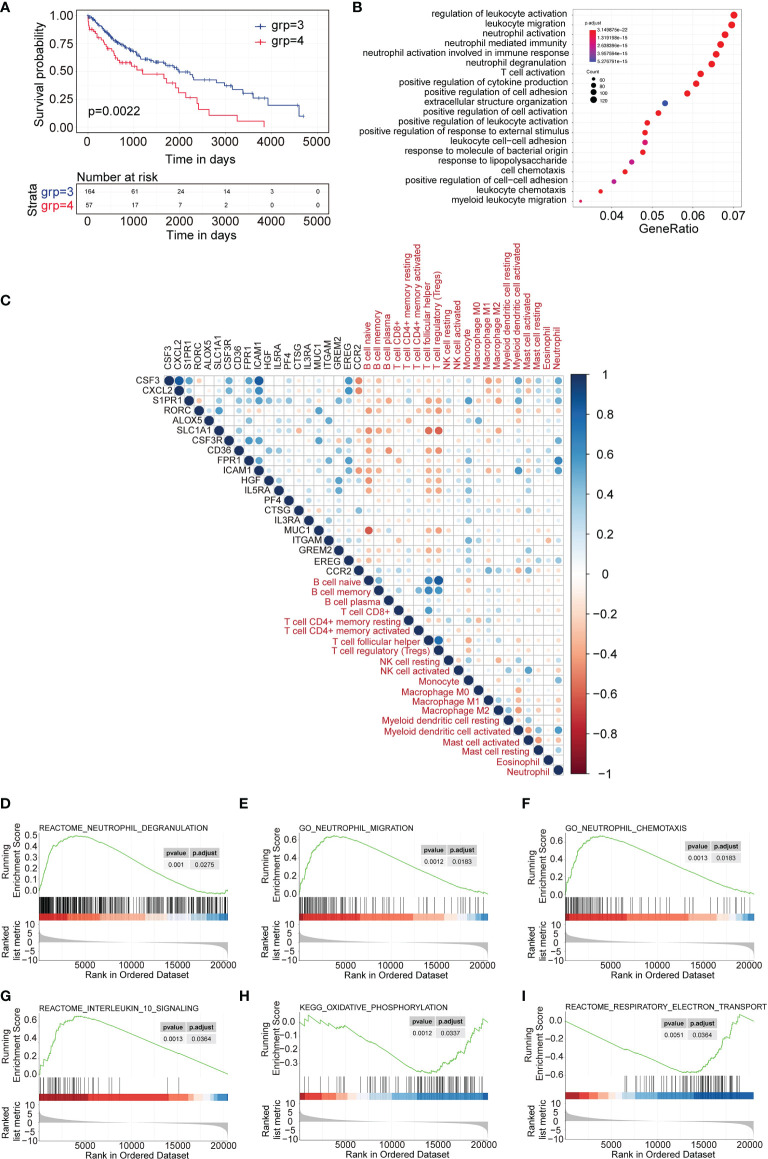
DEGs and differentially expressed cytokines identified between subtype 3 and subtype 4. **(A)** Survival analysis between subtype 3 and subtype 4. Kaplan–Meier curves showed the distinct outcome of patients in the two subtypes. **(B)** Gene Ontology analysis of upregulated genes in subtype 4 compared to subtype 3. The top 20 GO terms representing biological processes are shown. **(C)** Correlation plot of the correlation analysis between the expression of upregulated cytokines in subtype 4 and the scores of immune cell signatures. **(D–F)** GSEA representing the function of neutrophils of upregulated genes in subtype 4 compared to subtype 3. **(G–I)** GSEA representing the function of exhausted T cells of upregulated genes in subtype 4 compared to subtype 3.

Then we focused on the two differential subtypes—subtype 3 and subtype 4. First, we investigated the OS difference in TCGA-LUSC data. The data showed that subtype 3 had significantly better OS compare to subtype 4 (**Figure 4A**). Then, the differentially expressed genes between subtype 3 and subtype 4 were identified. There were 2,326 genes upregulated and 1,211 genes downregulated in subtype 4 compared to subtype 3. The GO showed that the upregulated genes were enriched with neutrophil activation, neutrophil-mediated immunity, T cell activation in terms of biological process (**Figure 4B**), and molecular function (**Supplementary Figure 2A**). The KEGG pathway enrichment showed that the top pathways were cytokine–cytokine receptor interaction, PI3K−Akt signaling pathway, chemokine signaling pathway, neutrophil extracellular trap formation, etc. (**Supplementary Figure 2B**).

Similarly, different cytokines between subtype 3 and subtype 4 were investigated. A total of 73 genes were upregulated (p < 0.05, and fold change >2) and 13 genes were downregulated in subtype 4 compared to subtype 3 (p < 0.05, and fold change <-2). Then the top 20 upregulated cytokine genes and 13 downregulated genes were used to evaluate the correlation index with immune cell infiltration scores for TCGA subtype 4 expression profiles. The data showed that most of the upregulated cytokines were positively correlated with neutrophils and monocytes but negatively correlated with B cells, follicular helper T cells, and regulatory T cells (**Figure 4C**). Most of the downregulated cytokines were positively correlated with B cells, follicular helper T cells, and regulatory T cells and negatively correlated with neutrophil cells (**Supplementary Figure 3**).

GSEA analysis showed that the upregulated genes were significantly enriched to neutrophil degranulation, neutrophil chemotaxis, and neutrophil migration (**Figures 4D–F**), which represented a correlation with neutrophil-related immune activity, while the GSEA analysis also showed that the upregulated genes were enriched with interleukin 10 signaling and oxidative phosphorylation, but not respiratory electron transport (**Figures 4G–I**). This indicated correlation with exhausted T cell function.

### Validation of the Risk Model in GEO Datasets

**Figure 5:**
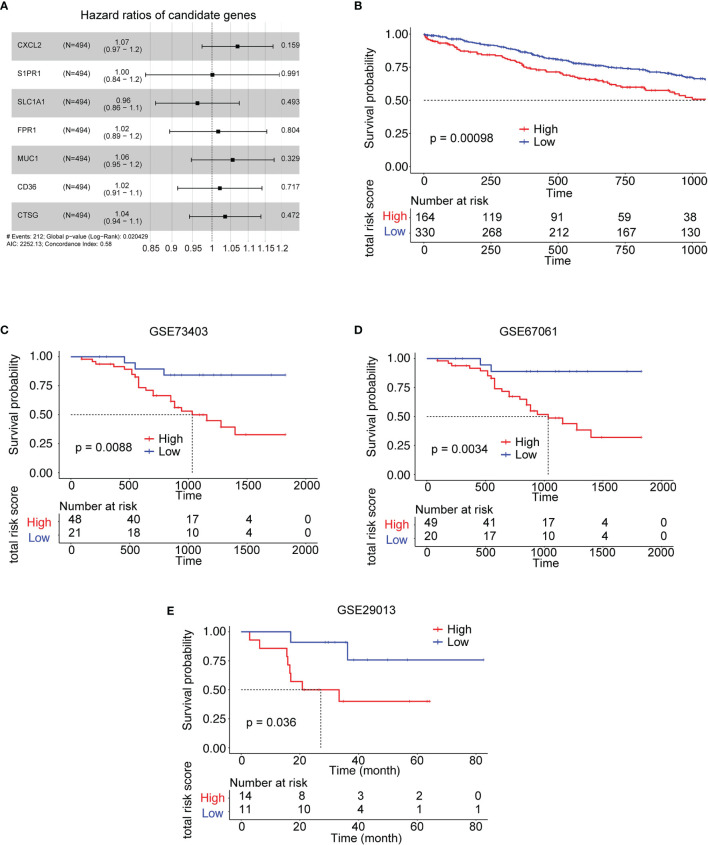
Kaplan–Meier survival analysis of the risk model gene signatures for both the training set and testing sets. **(A)** The forest plot for 7 genes used to construct the risk model. **(B)** The survival analysis of high- to low-risk patients in the training set TCGA LUSC. **(C–E)** The survival analysis of high- to low-risk patients in the validation sets from LUSC GEO datasets. #: note.

Based on the above results, 11 cytokine genes which were upregulated in subtype 4 and significantly correlated with follicular helper T cell or neutrophil were selected to construct the risk model. Lasso was used to perform variable selection to enhance the prediction accuracy with 7 genes selected, as shown in **Figure 5A**. TCGA expression data of LUSC were utilized as the training set to construct the risk model. There was a significant survival difference between low-risk subtype and high-risk subtype in the TCGA data set (**Figure 5B**) and three lung squamous cancer data sets from GEO datasets (**Figures 5C–E**).

### Validation of Immune-Related Gene *FPR1*

**Figure 6:**
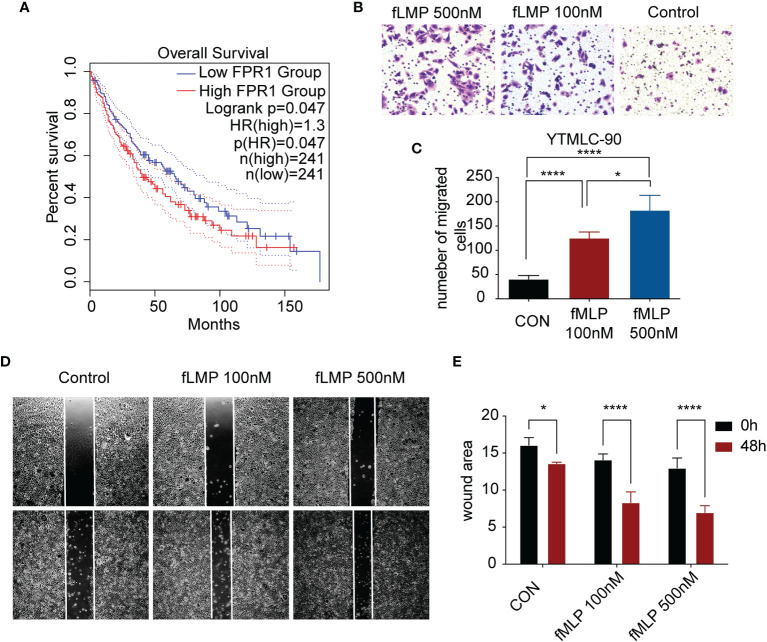
Validation of one of the risk model genes by experiment. **(A)** Kaplan–Meier survival analysis of high-low expression of *FPR1* in TCGA LUSC cohorts. **(B)** The images show Transwell experiments conducted in the YTMLC-90 cell line. **(C)** Quantification of cells migrating to the lower chamber in three different fields under a magnification of 400*. **(D)** Wound healing images of YTMLC-90 cells at 0 timepoint and 48 h by the indicated treatment. **(E)** The images were analyzed using ImageJ software to evaluate the scratch area by quantification of the areas occupied by the lesion. The graph shows the average values and the standard error of three experiments in triplicate. Each experiment was repeated three times, and the data are presented as the mean ± SD. *P < 0.05, ****P < 0.0001.

Among the 7 genes, *FPR1* was correlated with neutrophil significantly. A high expression of *FPR1* was associated with low OS in LUSC (**Figure 6A**). *FPR1* was originally found on human neutrophils (21). The activation of this receptor triggers many functions of neutrophils, including chemotaxis, degranulation, ROS production, and phagocytosis (22). The main ligands of *FPR1* are bacteria and mitochondrial formylated peptides (N-formylmethionyl-leucyl-phenylalanine, fMLP), which are effective polymorphonuclear leukocyte (PMN) chemokines. To explore its function in LUSC cells, LUSC cell line YTMLC-90 was utilized. Cells were treated with DMSO or 100 nM fMLP and 500 nM fMLP for the indicated duration time. The cell invasion and migration to the low chamber were significantly increased in the fMLP-treated group. The 500-nM-treated group had higher cell invasion and migration compared to the 100-nM fMLP-treated group (**Figures 6B, C**). Similarly, wound healing experiments also showed that the fMLP-treated group had higher migrated cells compared to the control group (**Figures 6D, E**). These results indicated that the activation of *FPR1* could increase the migration and invasion ability in LUSC cell lines.

## Discussion

Major advances and breakthroughs in human cancer immunotherapy have changed the treatment scheme of lung cancer (23). Due to differential response to ICI treatment, researchers have now focused more on the function of immune cell infiltration for the disease progression and overall prognosis of lung cancer (24). Thus, we analyzed immune infiltration differences in the TCGA LUSC cohort and identified four subtypes based on their immune-related gene expression profile. Four subtypes were identified, namely, subtype 1 (mixed type), subtype 2 (mixed type), subtype 3 (tumor enriched), and subtype 4 (immune enriched, fibrotic). These four subtypes showed distinct prognosis (**Figure 1**). In estimating the proportions of immune cell infiltration, CIBERSORT was used. The differences among four subtypes included macrophages, follicular helper T cells, neutrophils, and monocytes. M2 macrophages, neutrophils, and monocytes were most enriched in subtype 4 while least in subtype 3. Follicular helper T cells were least enriched in subtype 4 (**Figure 2**). The immune environment score was evaluated by the ESTIMATE approach. The biggest difference is between subtype 3 and subtype 4. Among the four subtypes, subtype 3 had the best OS while subtype 4 had the worst (**Figure 3**). Then, we focused on the two subtypes and discovered that the DEGs and differentially expressed cytokines were associated with exhausted T cells and neutrophils (**Figure 4**). Ultimately, we constructed a risk model by using 7 DEGs and validated its prediction value among the three independent GEO datasets (**Figure 5**) and validated one gene by using the experimental model (**Figure 6**).

Among the four subtypes, subtype 4 had the highest immune score and stromal score but lowest tumor purity score. However, opposite to subtype 4, subtype 3 had a low immune score and stromal score, but high tumor purity score. In addition, subtype 4 had a worse prognosis compared to subtype 3. Interestingly, compared to subtype 3, there was infiltration of more CD8 T cells. CD8+T cells are important in defense immunity against intracellular pathogens and tumors. In antitumor reactions, CD8+T cells are constantly exposed to antigens and inflammatory signals (25). In contrast, excessive and persistent signals lead to the state of T cell dysfunction, called “exhaustion.” Exhausted T cells are characterized as low proliferation and loss of effector function in response to antigen stimulation. A high expression of multiple inhibitory receptors such as PD-1, HAVCR2, and CTLA4 and metabolic alterations from oxidative phosphorylation to glycolysis are also markers of exhausted T cells (25, 26). Markers representing exhausted T cells were obtained from the literature (27). The results revealed that almost all these markers’ expressions were highest among four subtypes, while lowest in subtype 3 (**Figure 3**). GSEA analysis also showed consistent results that the upregulated genes in subtype 3 have negatively enriched oxidative phosphorylation (**Figure 4**).

The checkpoint receptors are expressed on activated immune cells to prevent overabundance (28). As for checkpoint gene expression, we found that the overall expression of checkpoint receptors was expressed higher in subtype 4 than in subtype 3, such as PD-1 and CTLA-4, resulting in an immunosuppressive environment. All these results pointed out that although subtype 4 is an immune-cell enriched environment, most infiltrated immune cells played a suppressive role.

The differentially expressed genes and cytokines between subtype 4 and subtype 3 both correlated with neutrophil activation. In cancer, neutrophils have emerged as an important component of the tumor environment (29). In most cancers, high neutrophil infiltration was associated with poor prognosis (30, 31). Studies showed that an elevated pretreatment neutrophil/lymphocyte rate was associated with shorter OS and progression-free survival and with lower response rates in patients with metastatic NSCLC treated with nivolumab independent of other prognostic factors (30). Neutrophils can be part of tumor-promoting inflammation by driving angiogenesis, extracellular matrix remodeling, metastasis, and immunosuppression. Conversely, neutrophils can also mediate antitumor responses by direct killing of tumor cells by participating in cellular networks that mediate antitumor resistance (32). *FPR1* is one of the important regulators of neutrophil recruitment (33). Studies have shown that *FPR1* plays an important role in inflammation, immunity, and tumors. Leslie et al. suggested that *FPR1* is an important regulatory molecule of neutrophils. In the pulmonary fibrosis model, *FPR1*–/– mice are exempt from bleomycin-induced pulmonary fibrosis. The mechanism is secondary to reduced neutrophil recruitment in the lung tissue in *FPR1*–/– mice after bleomycin stimulation (33). Shao et al. showed that in lung epithelial cells, the formylated peptides released from the mitochondria of damaged lung epithelial cells can stimulate migration of normal lung epithelial cells through *FPR1* and promote wound closure (34). Morris and other studies have shown that elevated levels of *FPR1* mRNA can predict the diagnosis of lung cancer, with a sensitivity of 55% and a specificity of 87% in the validation sample set (35). In our study, we also discovered that after activation of *FPR1*, the migration and invasion abilities of lung squamous cancer cells are increased, which is consistent with other studies. High expression of *FPR1* is correlated with poor prognosis of LUSC patients.

In conclusion, using the gene expression profile of global immune genes, we identified four subtypes in LUSC. Among them, two subtypes were distinct in terms of immunity features and immune checkpoint molecules. These directly correlate with patient outcomes. Given that subtype 4 is characterized as immune enriched but fibrotic, anti-PD1 or anti-PDL1 alone may not get an effective response. Combination treatment, chemotherapy plus anti-PD1, or anti-PDL1 or combined with anti-CTLA4 might be effective in these patients. These findings of the intra-tumor immune microenvironment may shed light on the strategy of immunotherapy in human lung squamous cancer.

## Data Availability Statement

The original contributions presented in the study are included in the article/**Supplementary Material**. Further inquiries can be directed to the corresponding author.

## Author Contributions

(I) Conception and design: LY, WZ, DP. (II) Administrative support: QZ. (III) Provision of study materials or patients: QZ. (IV) Collection and assembly of data: YaL. (V) Data analysis and interpretation: all authors. (VI) Manuscript writing: all authors. (VII) Manuscript revision: TC. (VIII) Final approval of manuscript: all authors. All authors contributed to the article and approved the submitted version.

## Funding

This study was supported by the Post-Doctor Research Project, West China Hospital, Sichuan University (No. 2020HXBH131); the National Science Foundation of China (No. 82103413); and the Technology Development Contract of West China Hospital of Sichuan University (HX-H2112309).

## Conflict of Interest

The authors declare that the research was conducted in the absence of any commercial or financial relationships that could be construed as a potential conflict of interest.

## Publisher’s Note

All claims expressed in this article are solely those of the authors and do not necessarily represent those of their affiliated organizations, or those of the publisher, the editors and the reviewers. Any product that may be evaluated in this article, or claim that may be made by its manufacturer, is not guaranteed or endorsed by the publisher.

## Supplementary Material

Supplementary Figure 1 Heatmap of the expression profiles of 19 checkpoint molecules among the four subtypes in the TCGA LUSC cohort. High expression is presented in red, and low expression is presented in blue.

Supplementary Figure 2
**(A)** Gene Ontology analysis of upregulated genes in subtype 4 compared to subtype 3. The top 20 GO terms representing molecular functions are shown. **(B)** KEGG analysis of upregulated genes in subtype 4 compared to subtype 3. The top 50 KEGG terms was shown.

Supplementary Figure 3 Correlation plot of correlation analysis between the expression of downregulated cytokines in subtype 4 and the scores of immune cell signatures.

Supplementary Table 1 SigClust analysis of four subtypes identified by the consensus cluster method.

Supplementary Table 2 Association of four molecular subtypes with clinical parameters in TCHA-LUSC cohort.
