# Supplementary figures and images for: Identification of Immune Subtypes of Lung Squamous Cell Carcinoma by Integrative Genome-Scale Analysis
Source: Front Oncol. 2022 Feb 2;11:778549. doi: 10.3389/fonc.2021.778549 (PMC8847157; doi:10.3389/fonc.2021.778549)

Supplementary Figure 1

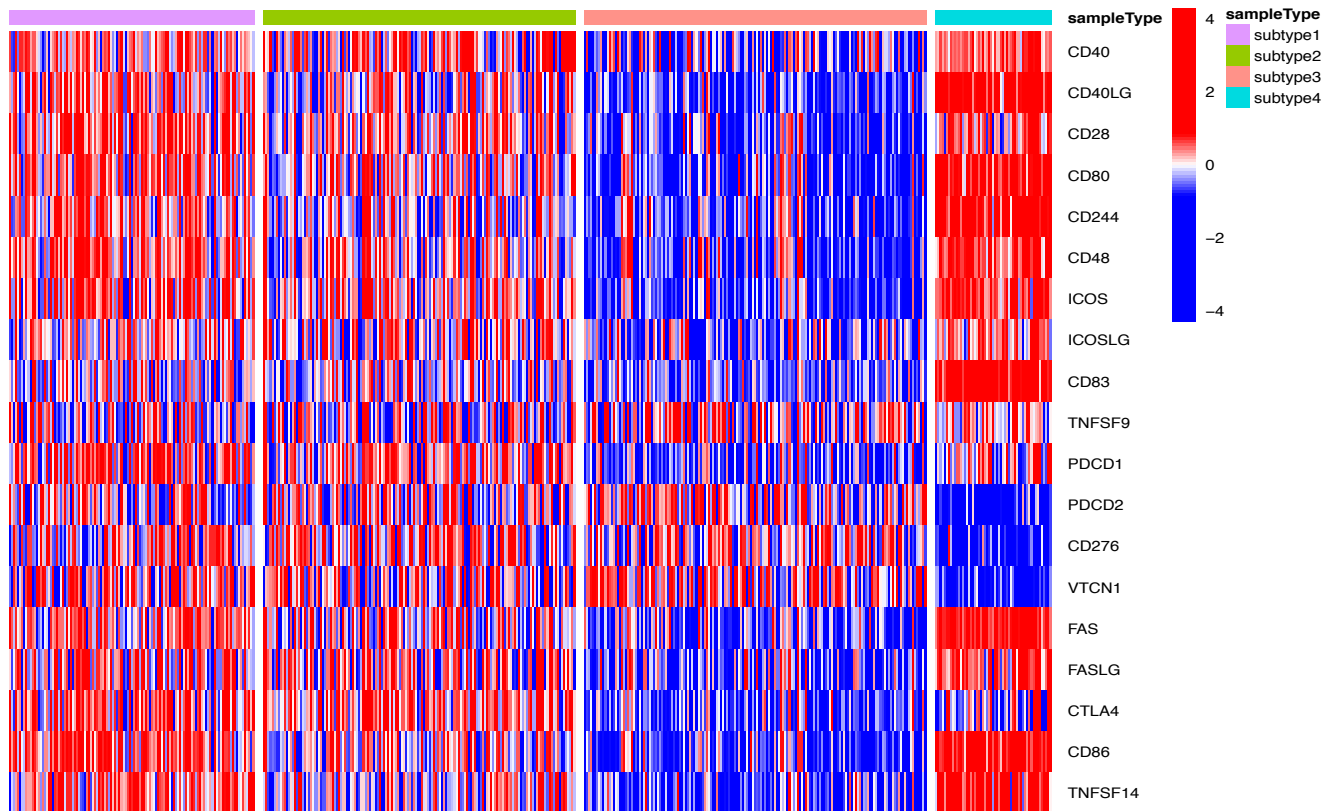

Supplement: Supplementary Figure 1 — Heatmap of the expression profiles of 19 checkpoint molecules among the four subtypes in the TCGA LUSC cohort. High expression is presented in red, and low expression is presented in blue. [file DataSheet_1.pdf]

Supplementary Figure2

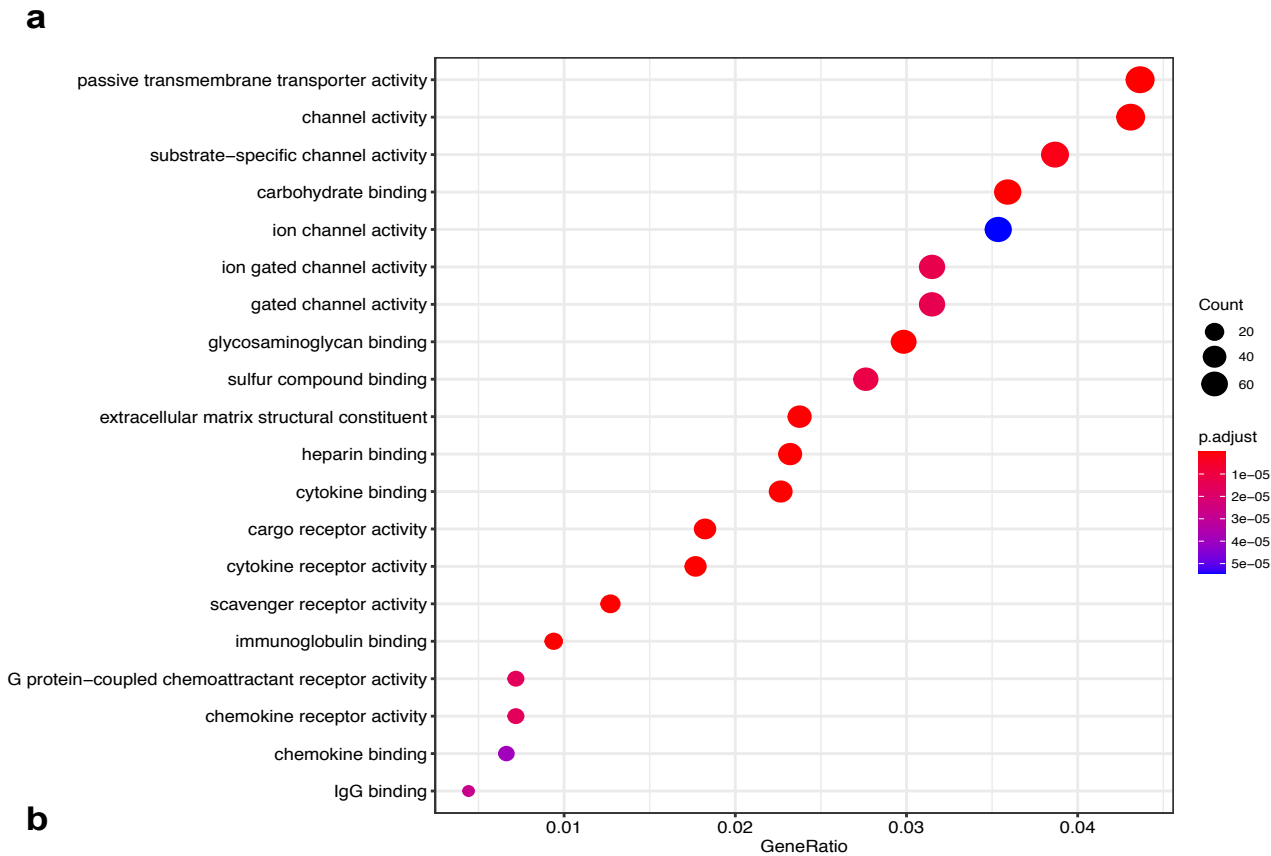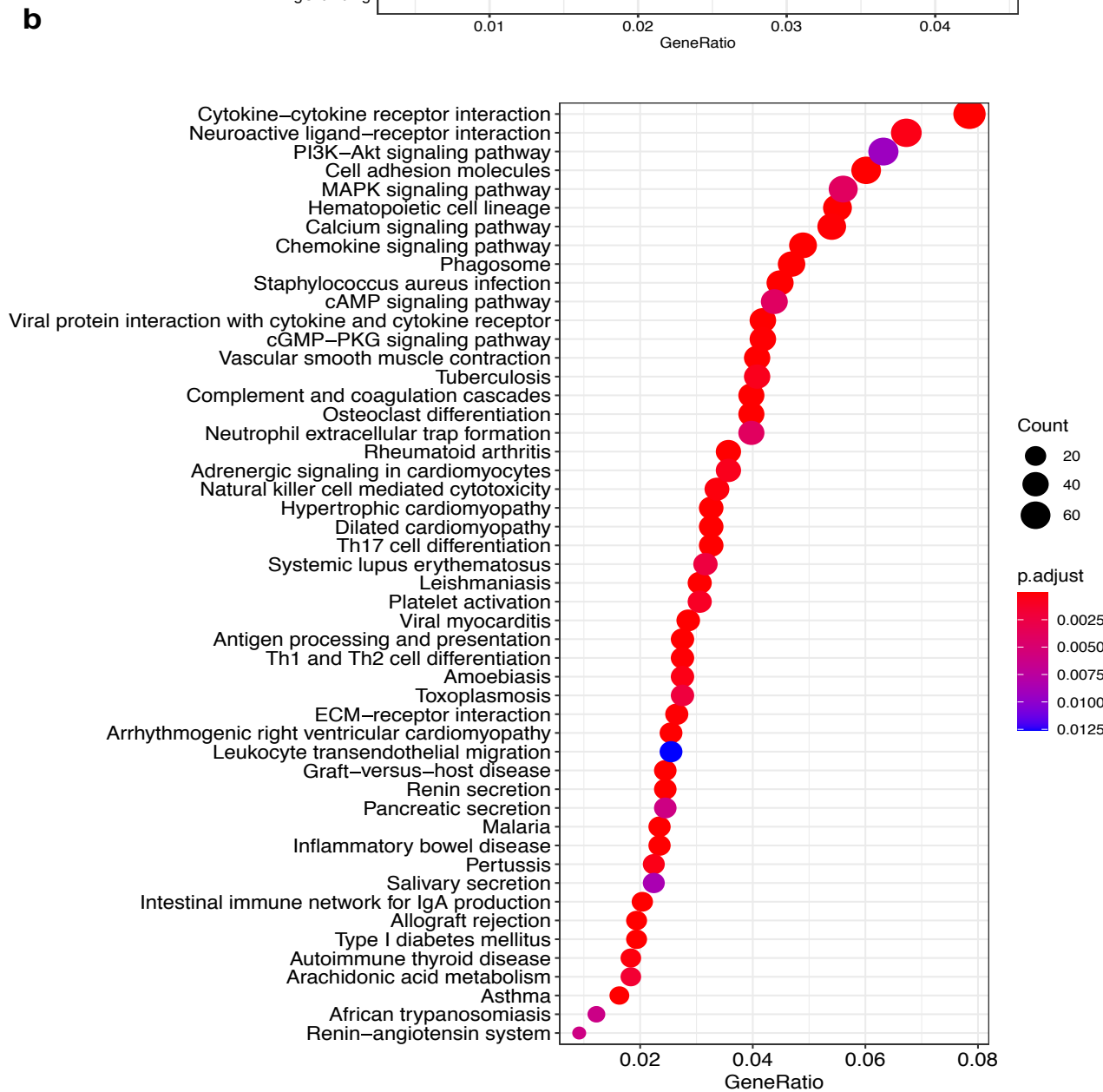

Supplement: Supplementary Figure 2 — (A) Gene Ontology analysis of upregulated genes in subtype 4 compared to subtype 3. The top 20 GO terms representing molecular functions are shown. (B) KEGG analysis of upregulated genes in subtype 4 compared to subtype 3. The top 50 KEGG terms was shown. [file DataSheet_2.pdf]

Supplymentary Figure 3

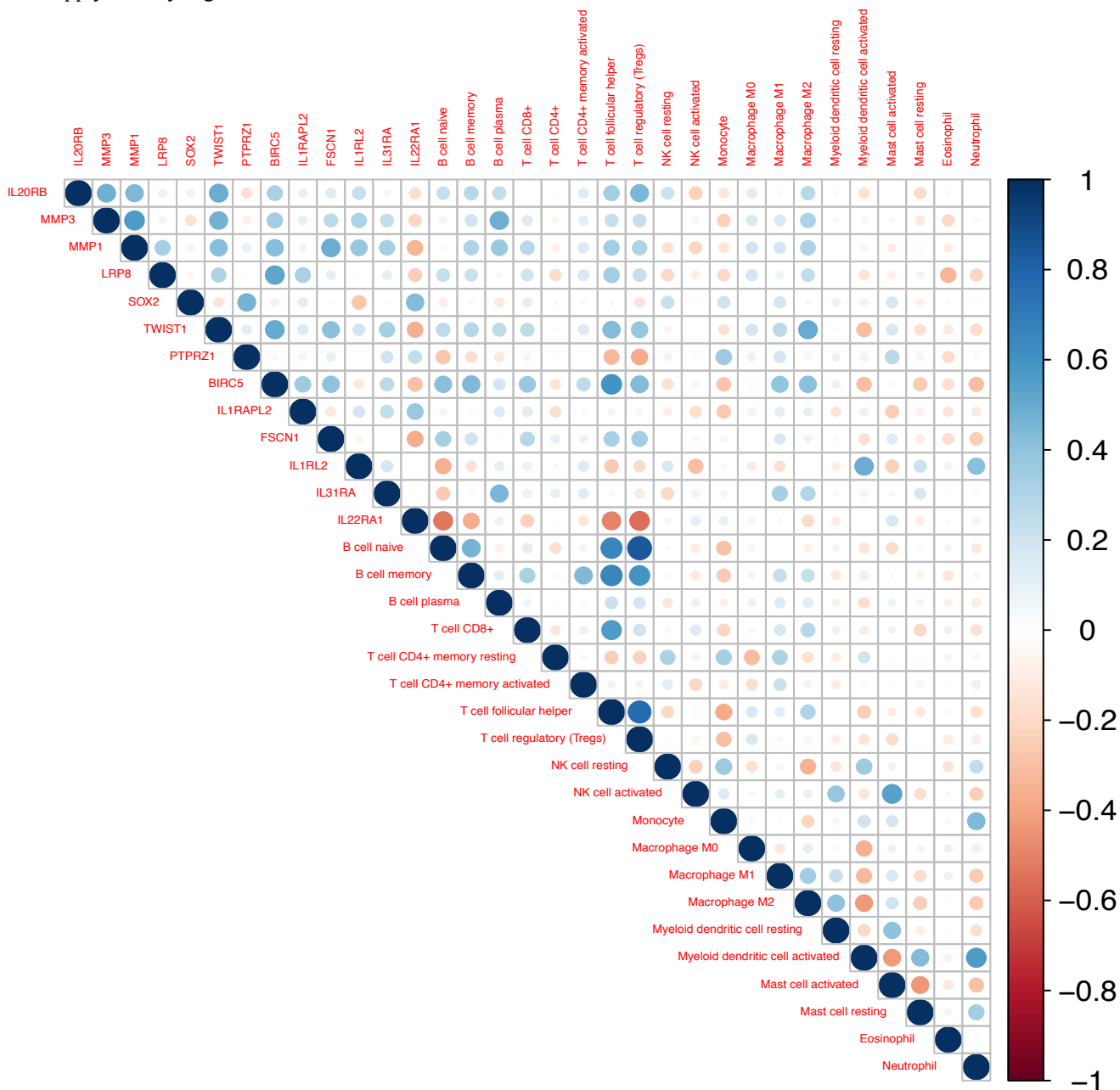

Supplement: Supplementary Figure 3 — Correlation plot of correlation analysis between the expression of downregulated cytokines in subtype 4 and the scores of immune cell signatures. [file DataSheet_3.pdf]
