# Supplementary material for: Identification of Immune Subtypes of Lung Squamous Cell Carcinoma by Integrative Genome-Scale Analysis
Source: Front Oncol. 2022 Feb 2;11:778549. doi: 10.3389/fonc.2021.778549 (PMC8847157; doi:10.3389/fonc.2021.778549)
Supplement: Supplementary Table 1 — SigClust analysis of four subtypes identified by the consensus cluster method. [file Table_1.docx]

Supplementary Table 1 P-values of pairwise comparison for four subtypes by SigClust analysis

| **compare** | **p-value** | **p.ajust** |
| --- | --- | --- |
| C1vsC2 | 0 | 0 |
| C1vsC3 | 0 | 0 |
| C1vsC4 | 0 | 0 |
| C2vsC3 | 0 | 0 |
| C2vsC4 | 0 | 0 |
| C3vsC4 | 0 | 0 |

Note: zero for p-values is only a computational approximation by the software.
