# Supplementary material for: Identification of Immune Subtypes of Lung Squamous Cell Carcinoma by Integrative Genome-Scale Analysis
Source: Front Oncol. 2022 Feb 2;11:778549. doi: 10.3389/fonc.2021.778549 (PMC8847157; doi:10.3389/fonc.2021.778549)
Supplement: Supplementary Table 2 — Association of four molecular subtypes with clinical parameters in TCHA-LUSC cohort. [file Table_2.docx]

| Clinical parameters |  | Molecular subtypes (n,%) | | | | *P* value |
| --- | --- | --- | --- | --- | --- | --- |
|  |  | subtype1 | subtype2 | subtype3 | subtype4 |  |
| Sex | Female | 39(32.2) | 38(24.7) | 37(21.9) | 16(28.1) | 0.24 |
|  | Male | 82(67.8) | 116(75.3) | 132(78.1) | 41(71.9) |  |
| Age | <60y | 15(12.4) | 26(16.9) | 40(23.6) | 10(17.5) | 0.17 |
|  | >=60y | 103(85.1) | 126(81.8) | 124(73.4) | 47(82.5) |  |
|  | NA | 3(2.5) | 2(1.3) | 5(3) | 0(0) |  |
| Stage | I | 72(59.5) | 64(41.6) | 78(46.1) | 30(52.6) | 0.13 |
|  | II | 30(24.8) | 61(39.6) | 52(30.8) | 19(33.3) |  |
|  | III | 17(14) | 23(14.9) | 37(21.9) | 7(12.3) |  |
|  | IV | 1(0.8) | 4(2.6) | 1(0.6) | 1(1.8) |  |
|  | NA | 1(0.8) | 2(1.3) | 1(0.6) | 0(0) |  |

Supplementary table 2 Association of four molecular subtypes with clinical parameters in TCGA-LUSC cohort.

Note; y, years; N/A, not applicable; *p* value is calculated by chi-square.
