# Supplementary material for: Identification of Immune Subtypes of Lung Squamous Cell Carcinoma by Integrative Genome-Scale Analysis
Source: Front Oncol. 2022 Feb 2;11:778549. doi: 10.3389/fonc.2021.778549 (PMC8847157; doi:10.3389/fonc.2021.778549)
Supplement: Supplementary file 6 [file DataSheet_4.docx]

RAW DATA

https://www.jianguoyun.com/p/Da3zjlkQyMvsCRjh4I0E
